# The Effect of the COVID-19 Pandemic on Pediatric Physician Wellness: A Cross-Sectional Study

**DOI:** 10.3390/ijerph19063745

**Published:** 2022-03-21

**Authors:** Joshua Belfer, Lance Feld, Sophia Jan, Joanna Fishbein, John Q. Young, Stephen Barone

**Affiliations:** 1 Pediatrics, Steven and Alexandra Cohen Children’s Medical Center, New Hyde Park, New York, NY 11040, USA; lance.feld212@gmail.com (L.F.); sjan1@northwell.edu (S.J.); sbarone@northwell.edu (S.B.); 2 The Feinstein Institutes for Medical Research, Northwell Health, Manhasset, New York, NY 11030, USA; jfishbein1@northwell.edu; 3 Medicine & Pediatrics, Donald and Barbara Zucker School of Medicine at Hofstra/Northwell, Hempstead, New York, NY 11549, USA; 4 Psychiatry, Donald and Barbara Zucker School of Medicine at Hofstra/Northwell, Hempstead, New York, NY 11549, USA; jyoung9@northwell.edu; 5 Zucker Hillside Hospital at Northwell Health, Glen Oaks, New York, NY 11004, USA

**Keywords:** medical education, trainees, pediatrics, COVID, burnout

## Abstract

The COVID-19 pandemic has provided challenges to all healthcare workers. While the brunt of treating COVID-19 patients fell upon adult providers, pediatricians also experienced significant stressors and disruptions. Academic pediatricians and trainees (fellows and residents) were redeployed to manage adult patients in hospitalist and intensive care settings and/or had major changes to their clinical schedules. In this study, we aimed to describe levels of self-reported depression, anxiety, and burnout in pediatric physicians following the initial wave of the pandemic at the largest integrated health system in New York State. A cross-sectional study was conducted among pediatric physicians who cared for patients during the COVID-19 pandemic within the Northwell Health System as part of the Northwell Wellbeing Registry, a longitudinal registry assessing the psychological impact of COVID-19 on healthcare providers. A total of 99 pediatric physician respondents were included in this study; 72% of whom were attendings, 28% of whom were trainees. Compared to attendings, trainees reported significantly higher proportions of burnout–emotional exhaustion (*p* = 0.0007) and burnout–depersonalization (*p* = 0.0011) on the Abbreviated Maslach Burnout Inventory. There was not a similar trend in probable depression or probable anxiety using the Patient Health Questionnaire. In a multivariable logistic regression model, being a trainee was significantly associated with increased odds of burnout–emotional exhaustion (OR 5.94, 95% Confidence Interval: 1.85–19.02). These findings suggest that fellows and residents were a vulnerable population during the COVID-19 pandemic. Training programs should pay special attention to their trainees during times of crisis, and future studies can help to identify protective factors to reduce the risk of burnout during these times.

## 1. Introduction

The impact of the initial wave of COVID-19 on the healthcare system in the spring of 2020 was substantial. No area of the United States was affected more deeply during this time than the New York metropolitan area [1]. As hospital administrators began to change their policies to address the growing patient burden, many groups recognized the need to protect trainees. The American Heart Association, in April 2020, published a statement which called on hospital administrators to “protect medical trainees on the COVID-19 frontlines, or do not send them in [2]”. Physicians and groups from many specialties, including both surgical and non-surgical, echoed the importance of protecting trainees, both physically and emotionally, from the hardships of the COVID-19 pandemic [3,4].

After reaffirming the Common Program Requirements for training programs [5], the Accreditation Council for Graduate Medical Education (ACGME) “granted a significant degree of flexibility” to programs “to realign their resident and fellow workforce to meet the increased clinical demands created by the pandemic [6]”. Specifically, the ACGME allowed programs to selectively enforce specific requirements based on a staging system related to the severity of the pandemic at each local institution [7]. As the initial months of the pandemic dragged on, groups began to look not only at the disruption on training caused by COVID but at the impact on mental health and wellbeing on trainees. These groups included both surgical and non-surgical subspecialties [8,9,10].

A severe strain was placed on healthcare facilities and clinicians that cared for adults. Many available resources were reorganized, and pediatricians, who already were facing unique challenges in the treatment of children with COVID-19, were redeployed into adult medical and intensive care units. While the full psychological impact of this experience has yet to be determined, the unique experiences of those pediatric providers who were redeployed to care for adults have begun to be chronicled.

Physician burnout is well documented in the medical literature, and concern about this has only been heightened due to COVID-19 [11,12,13,14]. Most of the literature on physician wellness in the time of COVID-19 has revolved around generalized wellbeing. There are only a few studies that have evaluated specifically trainee wellness. While some large studies have included pediatricians as part of a cohort, the data is limited when redeployment is specifically evaluated in a cohort strictly composed of pediatricians. In this study, we aimed to describe the proportions of pediatric physicians who self-reported symptoms of depression, anxiety, or burnout following a period of redeployment during the COVID-19 pandemic. Additionally, we aimed to identify demographic factors, including training status, associated with worse psychological outcomes.

## 2. Materials and Methods

This is a cross-sectional study to assess psychological outcomes among pediatric physicians who cared for patients during the COVID-19 pandemic within the Northwell Health System. Data utilized in this study were from the Northwell Wellbeing Registry, an ongoing, longitudinal registry assessing the psychological impact of COVID-19 on healthcare providers. The Northwell Health System is New York State’s largest integrated healthcare network, made up of 23 hospitals and over 830 outpatient facilities. An electronic, voluntary survey was sent to all physicians within the health system from June 21st to 21 August 2020. Eligible subjects included attending physicians, residents, and fellows who were employed or affiliated with the health system between 1 March and 31 May 2020. Informed consent and questionnaires were distributed via an electronic link to preserve anonymity. Emails were sent to attending physicians (*n* = 3560) and trainee physicians (*n* = 1888) that were directly employed by the health system, as well as affiliated/voluntary physicians (*n* = 7094) who were not employees of the health system. The total sampling frame consisted of 12,542 physicians, of which 620 completed the questionnaire and informed consent, for an overall completion rate of 4.9%. For this paper, a subanalysis was conducted only on responses from those who selected “Pediatrics” in response to the prompt “What is your area of specialty?” Study data were collected and managed using REDCap electronic data capture tools hosted at Northwell Health [15,16]. REDCap is a secure, web-based software platform designed to support data capture for research studies. This study was reviewed by an Institutional Review Board and was determined to be exempt as the data utilized in this study existed within an already approved registry.

Primary predictor variables include trainee status and redeployment status. Trainees were defined as those who self-identified themselves as “resident” or “fellow”, as compared to those identifying as “attending” physicians. Redeployment was defined as a physician being assigned to a role or setting that was different from their usual responsibility. Redeployed pediatric physicians were assigned to adult COVID-19 medical and intensive care teams.

Outcomes of interest included burnout, anxiety, and depression. Subscales from the 9-item modified Abbreviated Maslach Burnout Inventory (aMBI) were used to measure burnout-emotional exhaustion (EE) and burnout-depersonalization (DP) [17,18]. A modified Likert response scale was utilized due to a data collection error. The scale consisted of 0 = “Never”, 1 = “A few times a year”, 2 (3 in the original aMBI) = “A few times a month”, 3 (4 in the original aMBI) = “Once a week”, 4 (5 in the original aMBI) = “A few times a week”, 5 (6 in the original aMBI) = “Every day”, excluding the original 2 = “Once a month or less” option found in the original scaling. Validated cutoffs from previous studies (EE score ≥ 9, DP score ≥ 6) were used to indicate burnout–EE and burnout–DP, respectively.

Two items from the Patient Health Questionnaire (PHQ) were used to measure anxiety. Item responses ranged from zero to three and were summed to create an anxiety symptom score [19,20]. Probable anxiety was indicated for an anxiety symptom score greater than or equal to three. Two additional items from the PHQ were used to measure depression. Item responses ranged from zero to three and were summed to create a depression symptom score. Probable depression was indicated for a depression symptom score greater than or equal to three.

Workload changes were measured with the question “On a scale of 1–5, how do you feel about your clinical workload as compared to your typical workload since the pandemic started?” Responses were reclassified as a 3-level variable (1/2-much lighter/lighter workload, 3-similar workload, 4/5-heavier/much heavier workload). Disruption in training was assessed only to those who self-reported being a resident or fellow, and was measured with a question that asked, “On a scale of 1–5, how would you rate the disruption the pandemic caused in your workflow?” Item responses ranged from one (“There was no disruption in my workflow”) to five (“Complete disruption”).

Demographic information collected included gender, race, ethnicity, and marital status. Several questions assessed respondents’ feeling of support at work. The Brief Resiliency Scale score was also utilized to assess respondents’ ability to bounce back [21]. An adapted version of The Epidemic–Pandemic Impacts Inventory (EPII) for Healthcare Workers was utilized to assess tangible impacts of the pandemic across personal and social life domains [22,23]. The EPII is a novel tool constructed by a team of clinical and developmental psychologists who have expertise in the assessment of stress, trauma, resilience, and coping. The first 16 questions from the EPII were used as a surrogate score for COVID exposure. Additionally, several EPII questions were individually looked at inferentially. These included Q6 (Contact with distressed family members who cannot be with a loved one), Q13 (Insufficient support from workplace supervisors or administrators), Q22 (Feeling fearful of reprisal from coworkers, supervisors, or administration if you voice concerns about the safety of yourself, your coworkers, or your patients), Q23 (Feeling proud about what you have been able to accomplish at work), Q24 (Feeling as though you have made an important difference in patients’ lives), and Q25 (Feeling that you have grown as a worker or professional in this crisis).

Proportions of the above measures for burnout (EE and DP), anxiety, and depression were compared by demographics, trainee status, redeployment status, and EPII. Descriptive statistics (e.g., frequencies and proportions for categorical variables and means and standard deviations or medians and interquartile ranges for continuous variables) were computed. To compare categorical explanatory factors with each binary outcome, Fisher’s exact tests were performed. For continuous explanatory factors, Monte Carlo (MC) estimation for the exact Wilcoxon rank-sum test was utilized. Multivariable logistic regression modeling was used to further address the identification of potential demographic factors associated with worse outcomes for the sole outcome of Burnout–EE (due to the limited number of events in the other outcomes, multivariable modeling was not feasible). The multivariable model built for the outcome of burnout–EE forced trainee status and EPII sum score into the model. The reason for this is that trainee status was a main predictor of interest, and the EPII sum score was felt to be a strong confounder. Factors associated with Burnout–EE on univariate analysis at *p* < 0.1 were considered for inclusion in the final multivariable model (except for EPII Q6 or EPII Q13, as these were part of the EPII sum score, they were not considered as potential covariates in the final adjusted model). Due to the limited sample size, only one additional factor was considered for inclusion in the final model from the pool of the following factors: EPII Q22, EPII Q25, Partner/Marital status, Having/Feeling supported, and Brief Resilience Scale score. The final model was selected using a combination of the lowest Akaike Information Criterion (AIC) and greatest Area under the Curve (AUC). The final model included trainee status, the sum EPII score, and the additional question from the EPII on feelings of growth.

For all analyses, a result yielding *p*-value < 0.05 was considered statistically significant. Where appropriate, estimates along with their corresponding 95% confidence intervals are provided. All analyses were conducted using SAS version 9.4 (SAS Institute Inc., Cary, NC, USA).

## 3. Results

A total of 99 pediatric physician respondents were included in this study. Seventy-two percent of these respondents identified as attending physicians, while 28% identified as trainees. In total, 28% of respondents were redeployed between March and May 2020; 25% of attending physician respondents and 36% of trainee respondents were deployed. Sixty-seven percent of respondents were female, 75% were white, and 75% were married or engaged. Full demographic data of respondents can be seen in Table 1. The response rate for the overall registry was 4.9%.

For the entire cohort of respondents, 12% endorsed burnout via depersonalization, while 35% endorsed burnout via emotional exhaustion on the aMBI. On the PHQ tool, 7% endorsed probable depression and 15% endorsed probable anxiety. Regarding workload, 35% of respondents endorsed a lighter workload during the study period and 41% endorsed a similar workload, while 24% endorsed a heavier workload.

Comparing attendings to trainees, trainee status was associated with significantly higher proportions of burnout–EE (*p* = 0.0007) and burnout–DP (*p* = 0.0011). There was no significant difference in probable depression or probable anxiety according to trainee status. Trainee status was also associated with perceived workload changes, with more trainees reporting a similar workload and more attendings reporting a lighter workload (*p* = 0.0037). When asked about the disruption caused in training by the pandemic, 26% of trainees reported no or only mild disruption; 37% reported moderate disruption, while 37% reported significant or complete disruption to training.

In looking at the impact of redeployment to adult patient care services on psychological outcomes, redeployment was not found to be significantly associated with probable depression, probable anxiety, or either aspect of burnout. Those who were redeployed trended towards a heavier workload as compared to those who were not deployed, who trended towards a lighter workload; this trend approached but did not reach significance (*p* = 0.07). Gender, race, and ethnicity each were not found to be significantly associated with any of the outcomes studied (*p* > 0.05).

The utilization of clinical support resources was not associated with any psychological outcome. Similarly, the feeling of being supported was not associated with any outcome. Full cohort responses for tools assessing depression, anxiety, and burnout can be seen in Table 2 and Table 3.

For the final multivariable logistic regression model, being a trainee, after adjusting for EPII score (COVID exposure) and feeling of professional growth during the crisis, was significantly associated with increased odds of burnout-EE (OR 5.94, 95% Confidence Interval (CI): 1.85–19.02). Similarly, a higher sum EPII score was associated with increased odds of burnout EE (1.48, 95% CI: 1.19–1.84); for each single point increase in sum EPII score, the odds of burnout-EE increased by 48%. Looking at the additional EPII question Q25, answering “Not at all” to the question was shown to be associated with significantly greater odds of burnout-EE as compared to answering “Definitely”, while adjusting for other factors in the model (OR 12.5, 95% CI: 1.87–83.4). Similarly, answering “Somewhat” was also associated with significantly greater odds of burnout-EE as compared to answering “Definitely”, while adjusting for other factors in the model (OR 4.0, 95% CI: 1.20–13.45). Results can be seen in Table 4.

## 4. Discussion

To our knowledge, this is one of the first studies [24,25] that has evaluated physician, specifically trainee, wellness within a cohort of pediatricians associated with the COVID-19 pandemic. In a cohort of pediatric physicians affiliated with the Northwell Health System, burnout, depression, and anxiety were common among both trainees and attending physicians. A significantly higher proportion of pediatric physicians who identified themselves as trainees were identified as burned out on the Abbreviated Maslach Burnout Inventory as compared to attending physicians. A multivariable model revealed that being a trainee was significantly associated with increased odds of burnout per the single-item measure of emotional exhaustion. Neither redeployment nor clinical support was found to be associated with any psychological outcome evaluated.

The data regarding burnout in US physicians is worrisome. Recent studies have shown that the prevalence of burnout symptoms among trainees is similar to that of attending physicians (~49%) [26]. This is nearly double that of other US workers, highlighting a pervasive incidence in medicine today. Pediatricians are not immune from burnout. Some reports estimate that the proportion of pediatricians who suffer from burnout approaches 43% [27]. Interestingly, burnout rates among pediatricians may be linked to the higher prevalence of women in the field, who are more apt to experience career dissatisfaction, emotional exhaustion, and other significant symptoms indicative of burnout [27].

This study demonstrated that pediatric trainees were disproportionately affected, and experienced higher rates of burnout compared to attending pediatric physicians. The timing of when burnout originates during one’s medical career is important to consider. There have been several studies that have estimated that burnout peaks sometime during either residency or fellowship training, with a wide range of trainees experiencing physician burnout. A recent study in Pediatrics assessed the epidemiology of pediatric resident burnout across a three-year span, from 2016–2018 [28]. Each year, the prevalence of burnout exceeded 50%. The four identifiable risk factors that contributed most to burnout included fatigue, stress, work-life balance dissatisfaction, and recent medical error. Overall, this study was consistent with prior studies and highlights the significance of burnout in this population of medical providers.

Understanding the specific risk factors for burnout among pediatric trainees is important, and it appears that these aforementioned risk factors have seemingly played a similar role in the promotion of burnout as it relates to the COVID-19 pandemic. A French study evaluated burnout among pediatric trainees who had worked at a hospital affected by COVID-19 [24], with the prevalence found to be considerably lower than that which has been reported across US pediatric trainees [28]. Additionally, the study identified sex, number of hours worked per week, and anxiety (both before and during the pandemic) as risk factors associated with burnout. While this study was not designed to address burnout in multiple cohorts variably impacted by the pandemic, a study in the future might help to further differentiate COVID-19 related stressors in different contexts.

Further studies should assess additional factors that may have contributed to an increased proportion of burnout in trainees as compared to attending physicians. Items such as perceived comfort within the health system and perceived comfort within their specialty may have impacted their experience during the pandemic, and these areas should be evaluated in future studies. Additionally, it is unclear whether there are protective factors that contributed to those who were redeployed not experiencing significantly more burnout as compared to those who remained within their area of specialty. Powering the study using a larger, multi-institutional cohort could help to better control for confounders and would help to identify the impact of different factors with a more precise estimate.

There are several limitations to this study. As with any cross-sectional study, association does not equal causation. Since this is a cross-sectional survey by design, temporal relationships cannot be ascertained. The response rate for the overall registry was low (4.9%), and the small sample size for the pediatric subset is a limitation. It should be noted that the response rate of attendings as compared to trainees in the pediatric subset was comparable to that of the full cohort. Due to the small sample size, there is limited power to detect potentially small differences that may be clinically meaningful. Additionally, due to the lag of when responses were collected after the initial wave of the pandemic, caution in the results should be exercised due to recall bias. As such, the generalizability of results may come into question if the sample population was not representative of the target population. It should be noted that in the multivariable analysis, due to the small sample size, several findings had imprecision in the estimated odds ratio. While statistically significant, the odds ratios may have underestimated or overestimated the metrics assessed. Furthermore, there may be misclassification of the emotional exhaustion and depersonalization outcomes due to having one of the categories missing from the administered survey. Finally, the aMBI and EPII tools are not well-validated scales. However, the aMBI has compared favorably to the long-form version of the tool in the assessment of burnout [19]. The EPII, because it was developed to specifically assess the effects of COVID-19, has also not yet been fully validated. It has been used, however, in a number of recent publications to assess the toll that the pandemic has had on healthcare workers [29,30]. This study will add to the body of literature in this regard.

## 5. Conclusions

Trainee status was significantly associated with burnout–emotional exhaustion during the COVID-19 pandemic for pediatric physicians within the Northwell Health System. Neither redeployment nor clinical support was found to be associated with burnout, depression, or anxiety. Future studies can help identify specific contributors that increase the risk for trainee burnout during times of crisis.

## Figures and Tables

**Table 1:** Demographics of Pediatric Physician Respondents.

Demographic	*n* (%)
Sex	
Male	33 (33%)
Female	66 (67%)
Race	
White only	74 (75%)
Asian only	22 (22%)
Other/Multiple Races	3 (3%)
Marital Status	
Married/Engaged	74 (75%)
Single/Separated/Divorced/Widowed	25 (25%)
Level of Training	
Attending	71 (72%)
Resident/Fellow	28 (28%)
Redeployment Status	
Yes	28 (28%)
Attending	18
Resident/Fellow	10
No	71 (72%)
Attending	53
Resident/Fellow	18

**Table 2:** Survey Responses for Burnout *.

Measure	*n* (%)	*p*-Value
aMBI		
Burnout–EE, total cohort		
Yes	34 (35%)	
No	63 (65%)	
Burnout–EE, attendings		
Yes	17 (24%)	
No	53 (76%)	
Burnout–EE, trainees		
Yes	17 (63%)	
No	10 (37%)	
Burnout–EE, Attendings vs. Trainees		0.0007
Burnout–DP, total cohort		
Yes	11 (12%)	
No	84 (88%)	
Burnout–DP, attendings		
Yes	3 (4%)	
No	66 (96%)	
Burnout–DP, trainees		
Yes	8 (31%)	
No	18 (69%)	
Burnout–DP, Attendings vs. Trainees		0.0011

* Cronbach’s alpha value: aMBI—Emotional Exhaustion scale: 0.80, aMBI—Depersonalization scale: 0.72.

**Table 3:** Survey Responses for Probable Depression and Probable Anxiety *.

Measure	*n* (%)	*p*-Value
PHQ		
Probable Depression, total cohort		
Yes	7 (7%)	
No	91 (93%)	
Probable Depression, attendings		
Yes	6 (9%)	
No	64 (91%)	
Probable Depression, trainees		
Yes	1 (4%)	
No	27 (96%)	
Probable Depression, Attendings vs. Trainees		0.6693
Probable Anxiety, total cohort		
Yes	15 (15%)	
No	83 (85%)	
Probable Anxiety, attendings		
Yes	8 (11%)	
No	62 (89%)	
Probable Anxiety, trainees		
Yes	7 (25%)	
No	21 (75%)	
Probable Anxiety, Attendings vs. Trainees		0.1211

* Cronbach’s alpha value: PHQ—Depression scale: 0.84, for PHQ—Anxiety scale: 0.82.

**Table 4:** Multivariable Model for Burnout—Emotional Exhaustion.

Parameter		Beta Estimate	Standard Error	*p*-Value	Odds Ratio	95% CIs
Intercept		−4.839	1.045	<0.0001	0.008	(0.001–0.06)
Trainee	Resident/ Fellow vs. Attending	1.781	0.594	0.0027	5.94	(1.85–19.02)
EPII Sum Score		0.394	0.111	0.0004	1.48	(1.19–1.84)
Feeling that you have grown as a worker or professional in this crisis	Not At All (vs. Definitely)	2.526	0.968	0.0091	12.50	(1.87–83.36)
	Somewhat (vs. Definitely)	1.391	0.616	0.0240	4.02	(1.20–13.45)

## Data Availability

Data supporting reported results can be found in the Northwell Wellbeing Registry.

## STROBE Statement

Reporting for this study adheres to STROBE.
